# A Comprehensive Study of the Polypropylene Fiber Reinforced Fly Ash Based Geopolymer

**DOI:** 10.1371/journal.pone.0147546

**Published:** 2016-01-25

**Authors:** Navid Ranjbar, Mehdi Mehrali, Arash Behnia, Alireza Javadi Pordsari, Mohammad Mehrali, U. Johnson Alengaram, Mohd Zamin Jumaat

**Affiliations:** 1 Department of Civil Engineering, University of Malaya, 50603, Kuala Lumpur, Malaysia; 2 DTU Nanotech, Department of Micro- and Nanotechnology, Technical University of Denmark, 2800 Kgs. Lyngby, Denmark; 3 Department of Mechanical Engineering and Center of advanced Material, University of Malaya, 50603, Kuala Lumpur, Malaysia; Monash University, AUSTRALIA

## Abstract

As a cementitious material, geopolymers show a high quasi-brittle behavior and a relatively low fracture energy. To overcome such a weakness, incorporation of fibers to a brittle matrix is a well-known technique to enhance the flexural properties. This study comprehensively evaluates the short and long term impacts of different volume percentages of polypropylene fiber (PPF) reinforcement on fly ash based geopolymer composites. Different characteristics of the composite were compared at fresh state by flow measurement and hardened state by variation of shrinkage over time to assess the response of composites under flexural and compressive load conditions. The fiber-matrix interface, fiber surface and toughening mechanisms were assessed using field emission scan electron microscopy (FESEM) and atomic force microscopy (AFM). The results show that incorporation of PPF up to 3 wt % into the geopolymer paste reduces the shrinkage and enhances the energy absorption of the composites. While, it might reduce the ultimate flexural and compressive strength of the material depending on fiber content.

## Introduction

Geopolymers are inorganic aluminosilicate polymeric material which cure and harden at near ambient temperatures [1]. The production of geopolymer was accompanied by much lower carbon dioxide emission compared to ordinary Portland cement since their production does not need limestone calcination and fuel combustion in the kiln. Therefore, the term ecologically friendly ‘green’ cement is coined on it. On the other hand, due to their improved properties such as high early strength gain, durability against chemical attack, high surface hardness, and higher fire resistance, the interest in this cementitious material is increasing and they are now seen as an alternative to conventional Portland cement [2–6]. However, like most ceramics, it suffers from quasi-brittle characters, deficiency of low flexural strength and sudden failure [7–10].

Geopolymers show excessive shrinkage although lesser than Portland cement materials, it is a crucial factor and need to be considered [11–13]. Unlike Portland cement hydration, only a small amount of water known as “interstitial or structural water” has been incorporated into the geopolymer gel production; thus, this characteristic of geopolymer has led to less demand of mixing water [14]. However, a portion of free water is demanded to make the product workable and homogenous; this water evaporates at up to 150°C [11, 14, 15]. The free water lose can cause a large shrinkage deformation of specimens although different curing conditions influence the rate of shrinkage rate variation. A comparison of an alkali activated fly ash and a Portland cement based mortars indicated a high shrinkage stability of the fly ash based geopolymer and its less dimensional variation under two conditions of high humidity and laboratory environment due to the stability of the main reaction [16].

Incorporation of fiber into brittle matrix is an efficient method to enhance toughening mechanisms and flexural strength due to the controlling of crack propagation under different loading or environmental effects such as shrinkage [17]. Unlike most ceramics, a wide range of fibers, including organics, can be used as reinforcement in geopolymer since the synthesis temperature of the geopolymers are near ambient temperature [1]. Fiber-reinforced geopolymer composites were first investigated by Davidovits with the aim of fabricating molding tools and patterns for the plastics processing industry [18]. Subsequently, reinforcement of geopolymers with different type of fibers were carried on through organic fiber like cotton fiber [8] and protein- based fibers [19], carbon fibers [17, 20], steel fibers [7, 21] and polyvinyl alcohol (PVA) fibers [22] to overcome the brittleness and catastrophic failure of the matrix.

Polypropylene fiber (PPF) have been extensively used as a reinforcement in Portland cement based materials because of its high toughness and durability; while there was a conflict about the correlation of the PPF content and the corresponding compressive strength of the concrete. Although it was stated by Building Research Establishment (2000) that incorporation of PPF reduces the compressive strength of concretes significantly, some others reported that there was no tangible reduction on compressive strength because of the PPF content [23–25]. This conflict was studied by A. Richardson and he was concluded because of the cement bond breaking by PPF, the compressive strength of the concrete is reduced notebly [26]. Although the similar reduction in strength was expected in geopolymers by inclusion of PPF, it was reported that early compressive strength of fly ash/calcined kaolin geopolymer increased at 1 and 3 days to about 68% and 20%, respectively, by addition of 0.5 wt% PPF into matrix. Moreover, the early flexural strength of the composite including 0.75% PPF was increased about two fold for both days [27]. Why polypropylene did not have such an adverse effects, that reported by A. Richardson [26], on geopolymers was the main objective of this research. Therefore, in this study PPF is characterized firstly; then, the low and high volume of PPF (0.5%, 1%, 2%, 3%, 4%, and 5%) was incorporated in fly ash based geopolymer and mechanical properties and shrinkage of the corresponding composites were measured up to 56 days to cover the short and long term effects of the fiber. Moreover, variation of slump and setting time were determined to evaluate the fresh properties of the materials. It was observed that mechanical behavior of the composite was influenced by the adverse effect of shrinkage over time and the weak bond between PPF and geopolymer matrix. While, flexural toughness was increased and mode of failure changed from brittle to ductile in high PPF content fly ash based geopolymer composites.

## Materials and Testing Methods

### 2.1 Geopolymer precursor characterization

**Fig 1:**
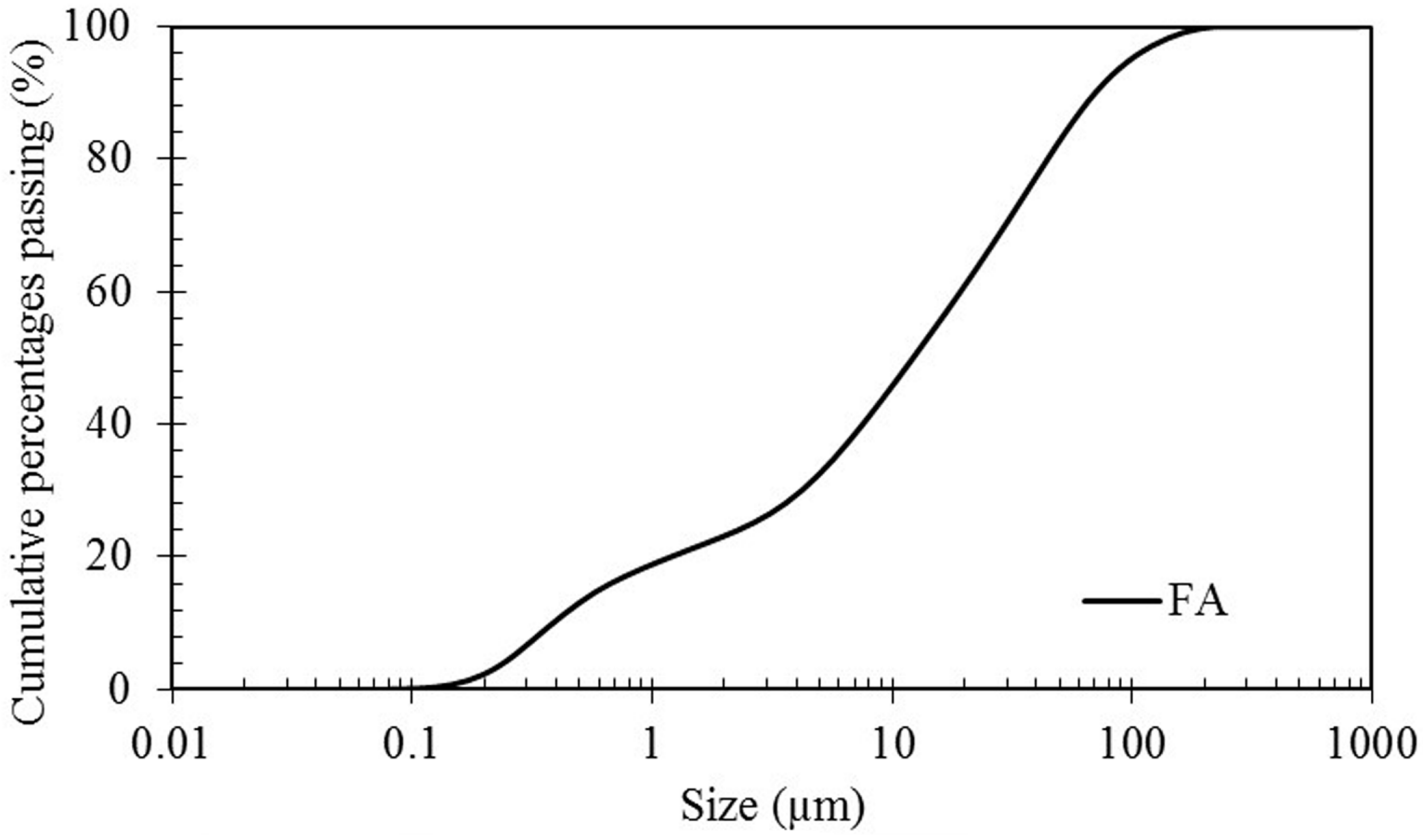
Particle size distribution of the fly ash.

Class F fly ash was obtained from local industry, Lafarge Malayan Cement Bhd of Malaysia. The chemical composition of the fly ash used in this research as determined by X-ray florescence (PANalytical Axios mAX instrument) was shown in Table 1. The particle size analysis was performed by Mastersizers Malvern Instruments and shown in Fig 1; moreover, median particle size and specific gravity of the fly ash are 12.19 μm and 2.18.

**Table 1:** Chemical composition of the fly ash.

Composition	Fly ash (%)
SiO_2_	75.8
Al_2_O_3_	15.9
Fe_2_O_3_	3.9
K_2_O	1.1
TiO_2_	1.0
CaO	0.9
SO_3_	0.3
MgO	0.3
P_2_O_5_	0.2
Na_2_O	0.2
ZrO_2_	0.1
MnO	0.1

Multifilament PPF was prepared from Timuran Engineering SDN BHD (Malaysia) which consisted of a straight individual fiber 12.19 mm in length, ~40 μm in diameter, 0.9 specific gravity, tensile strength of about 310 to 414 MPa and Young's modulus 345 MPa to enhance the toughening mechanism of the geopolymer matrix.

Alkaline activators in the investigation consisted of sodium silicate and sodium hydroxide solutions. Sodium hydroxide (NaOH) was prepared in pellet form with 99% purity while sodium silicate (Na_2_SiO_3_) was used in liquid form with about 1.5gr water per millilitre at 20°C with a modulus ratio of 2.5 (SiO_2_/Na_2_O, SiO_2_ = 30% and Na_2_O = 12%).

### 2.2 PPF fly ash based geopolymer composite preparation

16 molar NaOH was mixed with Na_2_SiO_3_ with the ratio of 0.4. The mixture was diluted with extra water in order to make the geopolymer paste workable and get a mass ratio of Na_2_SiO_3_:NaOH:H_2_O of 2.5:1.0:0.7 after mixing with the fly ash [28]. PPF was added to the alkali activator and stirred in order to make the uniform suspension and to overcome the poor distribution. The mixture was added to the fly ash gradually with a solution to solid ratio of 0.5 and mixed for 5 minutes; the material was immediately poured into stainless steel molds and cured in a 65°C Memmert ULM600 oven for 24 hours. Afterward, the molds were dismantled and specimens kept in ambient condition with an average temperature and humidity of 32°C and 65%, respectively, until the day of testing. It is notable that, according to our preliminary experiments, dry mixes of PPF and fly ash will result in accumulation of branch of multifilament fibers in particular place, non-uniformity and agglomeration of the matrix. The PPF content in geopolymer paste varied in the range of 0.5%, 1%, 2%, 3%, 4%, and 5%.

### 2.3 Flow measurement test

Flow measurement test were conducted in accordance with ASTM C1437-13 in order to evaluate the workability of the geopolymer composites [29]. For this measurement, the geopolymer paste specimens were prepared by 400 g of the fly ash with the same mix design as mentioned in section 2.2. Immediately after 5 min mixing, the paste was poured into the truncated conical mold (top diameter = 70 mm, bottom diameter = 100 mm, height = 50 mm) in two equal layers and tamped 20 times for compaction. After a minute, the truncated cone was lifted up and immediately the specimen was tamped 25 times in 15 seconds. The flow (S) is the result of increase in base diameter of the paste (D), expressed as a percentage of the original based bottom diameter (D0) by the following equation:

$$S=\left(\frac{D-D0}{D0}\right)\times 100$$

### 2.4 Setting time

Setting time test is performed adjacent to air based on ASTM C191–13 [30]. However, this test had to be optimized by covering the surface of the geopolymer samples with a thin layer of engine oil to avoid the evaporation of water and fast hardening of the crust adjacent to hot air. The sample was kept in 65°C oven. The penetration of Vicat needle was recorded every 10 minutes.

### 2.5 Shrinkage measurement

**Fig 2:**
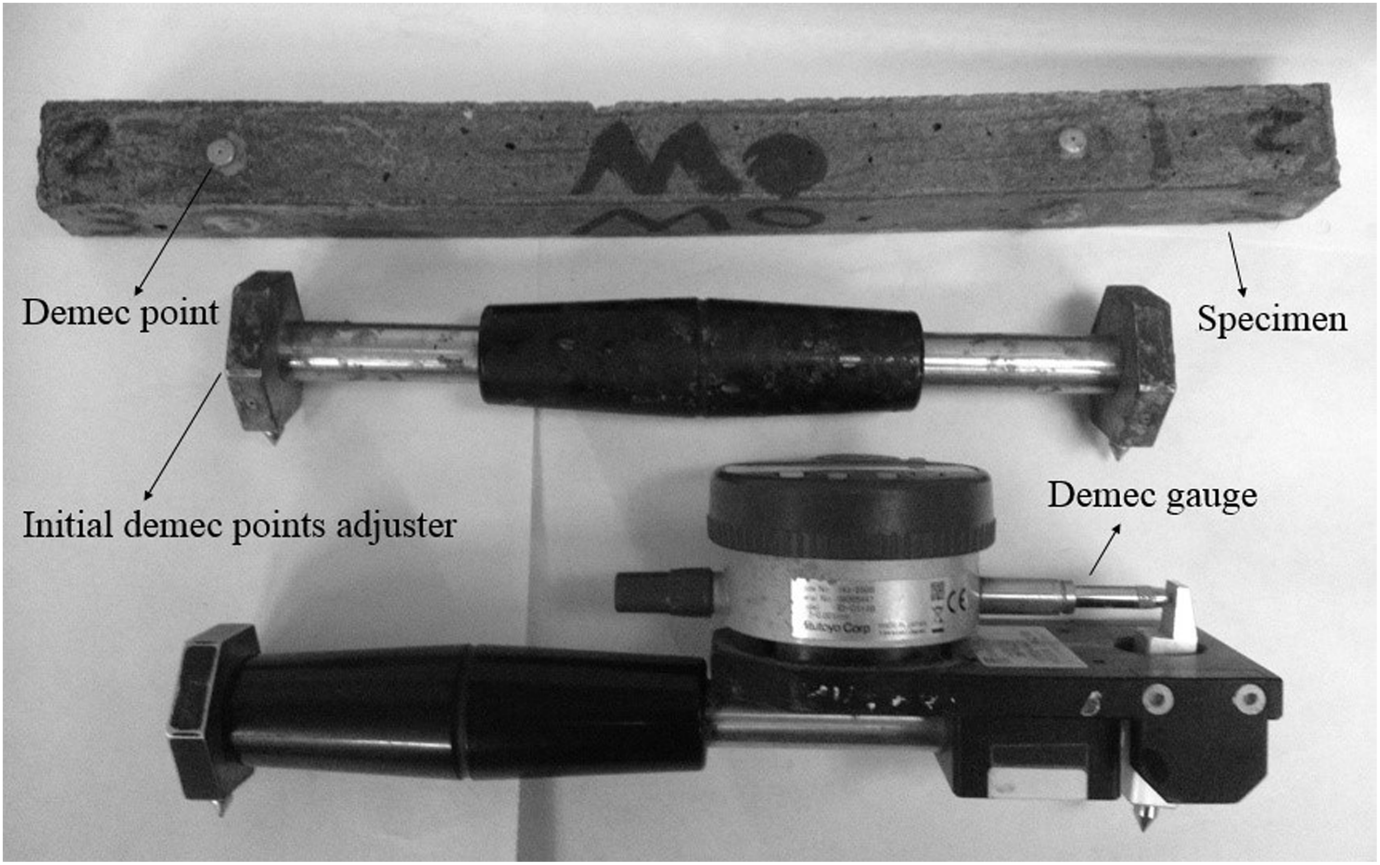
Shrinkage measurement equipment.

The shrinkage specimens were prepared on 25x25x300mm prisms. After 24 hours hot curing at 65°C, they were taken out and demec points were attached to the surface of the specimens by using Araldite 5-Minute AB Epoxy Adhesive. The variation of shrinkage over time was measured by using of Mitutoyo Absolute Digimatic Indicator ID-C112B apparatus with the range and resolution of 12.7 mm and 0.001 mm, respectively. Fig 2 indicates the shrinkage measurement tools of this study.

### 2.6 Density

The value of bulk density was determined by Archimedes method in accordance with ASTM C-20 after 60 days [31]. To prevent crack formation due to drying, the saturated and suspended on density measurement weights were carried out before the dried one. The saturated and suspended weight were measured after 2 hours boiling followed by immersing specimens under ionized water in vacuumed condition for 24 more hours. Then the samples were kept in 105°C oven condition for 24 hours to remove the water and obtain the dry weight.

### 2.7 Mechanical properties testing

The flexural strength development of the specimens was obtained after 2, 7, 14, 28, 56 days; 25x25x100mm prisms were prepared for three point bending with the rate of 0.2 mm/min and span of 75mm. However, variation of compressive strength was measured in 7 and 56 days on 25mm cubes with the displacement control rate of 0.5 mm/min; INSTRON-3369 machine was used to determine all the mechanical properties. Nominal flexural toughness of the material was reported as the area under flexural strength-deflection of each specimens; the calculation were done based on the flexural displacement of first crack “δ” for 3δ, 5.5δ, 10.5δ and 15.5δ in accordance to the δ suggested in ASTM C1018-97 [32]. Moreover, crack propagation and toughening mechanism of PPF geopolymer composite were recorded by a Dino-Lite digital microscope with magnification of 10x~50x and 200x.

### 2.8. Microstructure analysis

Field emission scan electron microscopy (SEM, Quanta FEG 450- Netherlands) was used to determine the texture of PPF, micro structure of the fly ash based geopolymer, the micro cracks and the interfacial transition zone of the PPF and geopolymer matrix. An atomic force microscope (AFM) (Bruker Nano, Santa Barbara, USA) fitted with a silicon-nitride cantilever (SNL-10, Bruker), controlled by the software Nanoscope III, was used in the scan-assist mode to measure the topography of the fibers surface at a nanometric scale. A single fiber was located on double-side tape which was covering the sample holder. Images with the size of 3.6μm × 3.6μm were obtained. Fourier transform infrared spectroscopy (FTIR) analysis were carried out using a Perkin Elmer System series 2000 spectrophotometer in a frequency range of 4000–400 cm-1 to identify the functional group of the PPF. X-ray diffraction (XRD) analysis were performed on an Empyrean PANALYTICAL diffractometer with monochromated Cu Kα radiation (λ = 1.54056 Å), operated at 45 kV and 40 mA with a step size of 0.026 deg and a scanning rate of 0.1 deg s^-1^ in the 2θ range of 5 to 75 deg to verify the change in crystalline phases of fly ash based geopolymer because of the PPF incorporation.

## Result and Discussion

### 3.1 PPF characterization

#### 3.1.1 Physical properties of PPF

**Fig 3:**
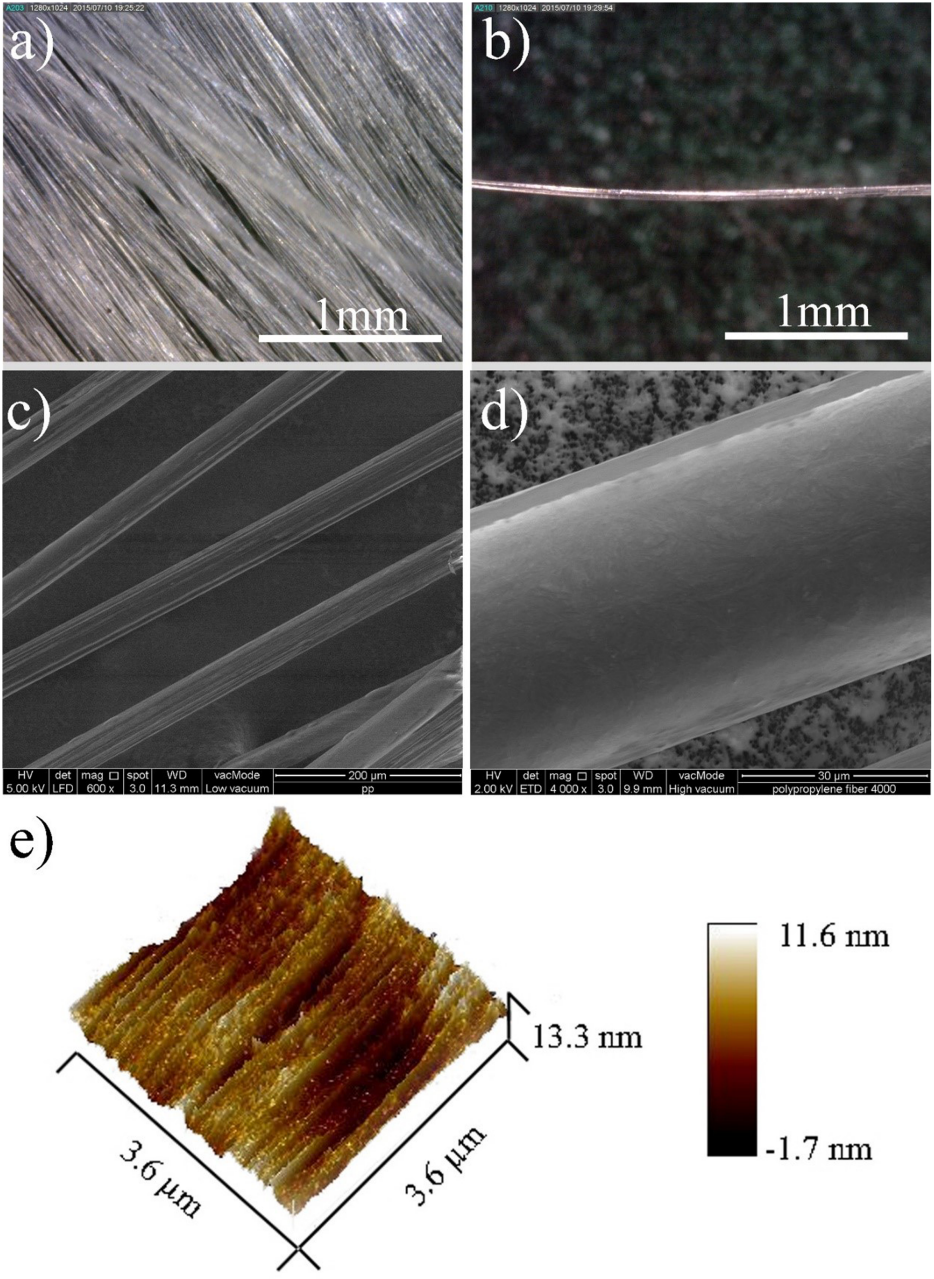
(a) Multifilament and (b) individual PPF determined by optical microscope camera, (c) and (d) FESEM images of the PPF and (e) AFM of the fiber texture.

Fig 3a and 3b show the images of multifilament and individual PPF, respectively, obtained by optical microscope camera. When the multifilament PPF stirs in alkali activator solution, they separates to a uniform distributed individual fiber mixture. The smooth texture with small corrugation on the surface of the PPF is shown in Fig 3c and 3d. The height of the fiber corrugations as determined by AFM was about ~13 nm and shown in Fig 3e.

#### 3.1.2 Functional groups in PPF

**Fig 4:**
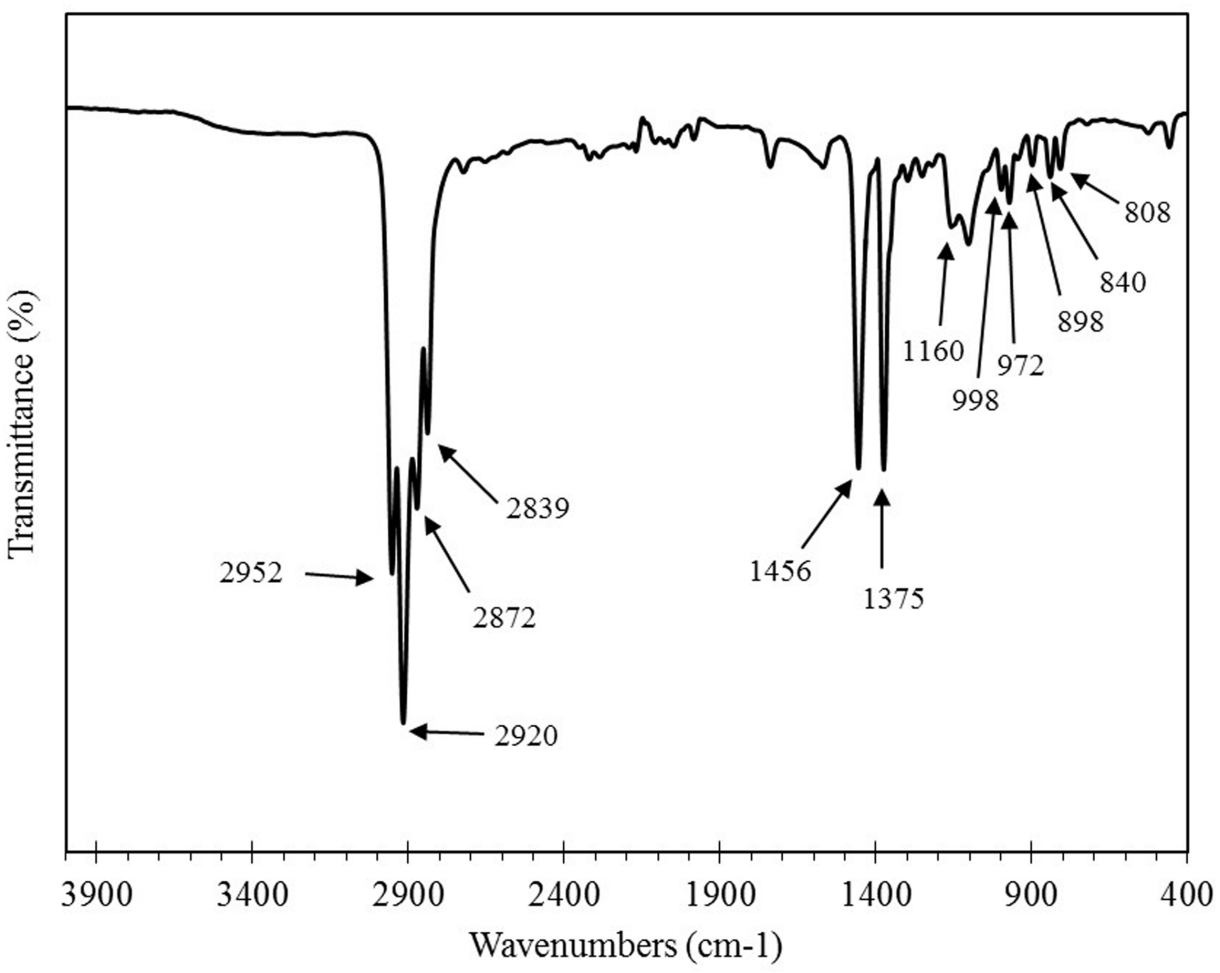
Fourier transform infrared spectra of polypropylene.

ATR-FTIR spectroscopy was performed on the PPF fibers in order to investigate the functional groups of these fibers, Fig 4. Accordingly, the ATR-FTIR spectrum of PPF fibers showed four large peaks in the wavenumber range 2800–3000 cm^-1^: the peaks at 2952 and 2872 cm^-1^ were allocated to the CH_3_ asymmetric and symmetric stretching vibration respectively, while the peaks at 2920 and 2839 cm^-1^ were attributed to CH_2_ asymmetric and symmetric stretching vibrations respectively. The spectrum also showed two intense peaks at 1456 and 1375 cm^-1^: the peak at 1456 cm^-1^ was due to CH_3_ asymmetric deformation vibrations (or CH_2_ scissor vibrations), whereas the peak at 1375 cm^-1^ was caused by CH_3_ symmetric deformation vibrations. The ATR-FTIR spectrum of PPF fibers also showed number of smaller peaks at wavenumber range 800–1200 cm^-1^: the peak at 1160 cm^-1^ was attributed to C-C asymmetric stretching, CH_3_ asymmetric rocking and C-H wagging vibrations, the peak at 998 cm^-1^ was due to CH_3_ asymmetric rocking vibrations, the peak at 972 cm^-1^ was assigned to CH_3_ asymmetric rocking and C-C asymmetric stretching vibrations, the peak at 898 cm^-1^ was allocated to CH_3_ asymmetric rocking and C-C asymmetric and symmetric stretching vibrations, and at last, the peaks at 840 and 808 cm^-1^ were caused by CH_2_ rocking vibrations [33].

### 3.2. X-Ray Diffraction analysis

**Fig 5:**
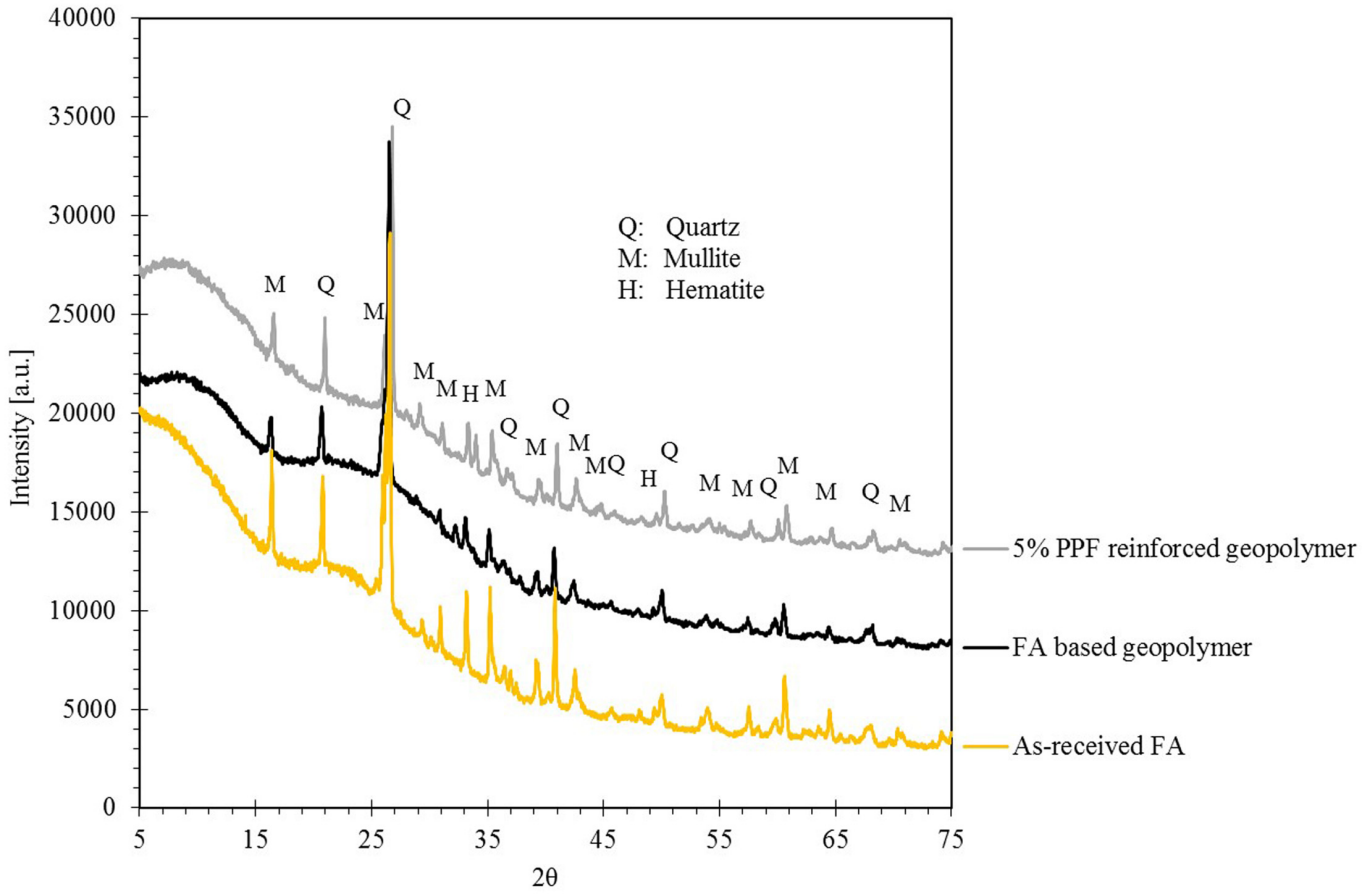
XRD patterns of the fly ash based geopolymer and 5% PPF reinforced FA based geopolymer.

Fig 5 shows the XRD patterns of the as-received fly ash, the fly ash based geopolymer without PPF reinforcement and the 5% PPF reinforced composite. As observed, FA based geopolymer mortar consisted of main crystalline phases of quartz and mullite which are originating from fly ash. When the FA react with alkali activators, its amorphousness is reduced and consequently the crystalinity of the product increased. Furthermore, the XRD analysis confirmed that the incorporation of PPF in to FA based geopolymer did not cause formation of other crystalline phases in the composite.

### 3.3. Fresh properties of the matrices

**Fig 6:**
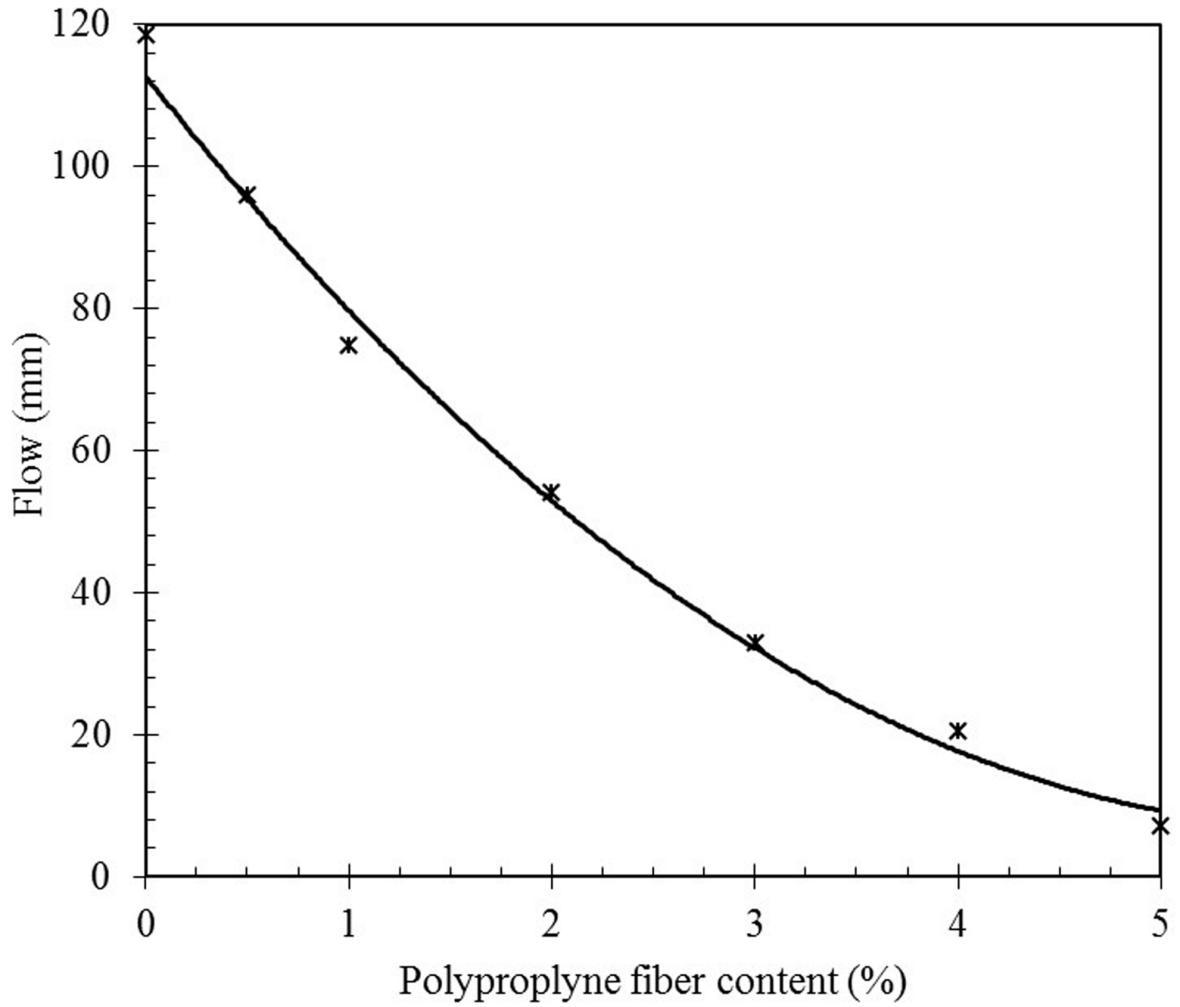
Influence of PPF on slump of the geopolymer composite.

Fig 6 shows the influence of the PPF content on the flow reduction of the geopolymer composites in fresh state. As observed, the flow of pure geopolymer paste is relatively high and tends to flow by gravity. Addition of low fraction, 1–3% of fibers into geopolymer paste seems harsh when static though the stiffening effect of the fibers tends to disappear under vibration. However, general addition of fiber offers higher shear resistance to flow which results in a decrease of flowability. Higher polypropylene content specimens up to 4% and 5% tend to keep their mold shape with very low workability, placeability and compaction.

**Fig 7:**
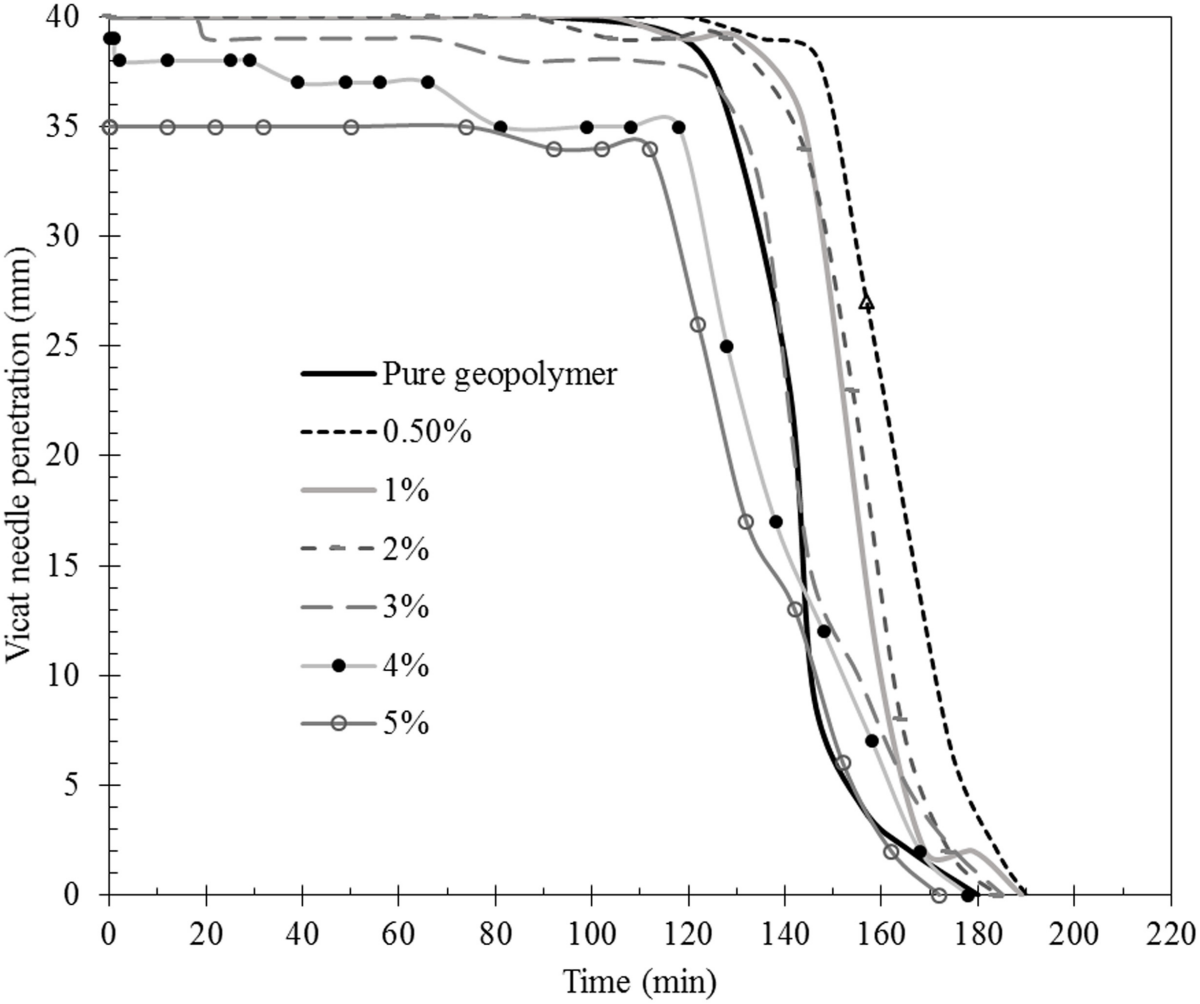
Influence of the polypropylene fibers on setting time of geopolymer composite.

Fig 7 showed the setting time of different fraction of PPF reinforced geopolymer matrices. As indicated, addition of low fraction of polypropylene into geopolymers delayed the initial and final setting of the matrixes which was the highest in the case of 0.5% content. However, further increase in PPF content reduced the initial setting time. Noteworthy, an abnormal needle penetration measured in the case of high PPF content of 4% and 5% matrixes. Vicat needle could not penetrate completely through the specimens due to the accumulation of fibers at the tip of needle once in contact with the net of fibers, thus stopping the needle. Further, the final setting time was increased by increasing the PPF content. This might be attributed to the porous structure of the material and the low thermal conductivity of the air trapped in pores and the incorporated PPF.

### 3.4. Bulk density

**Fig 8:**
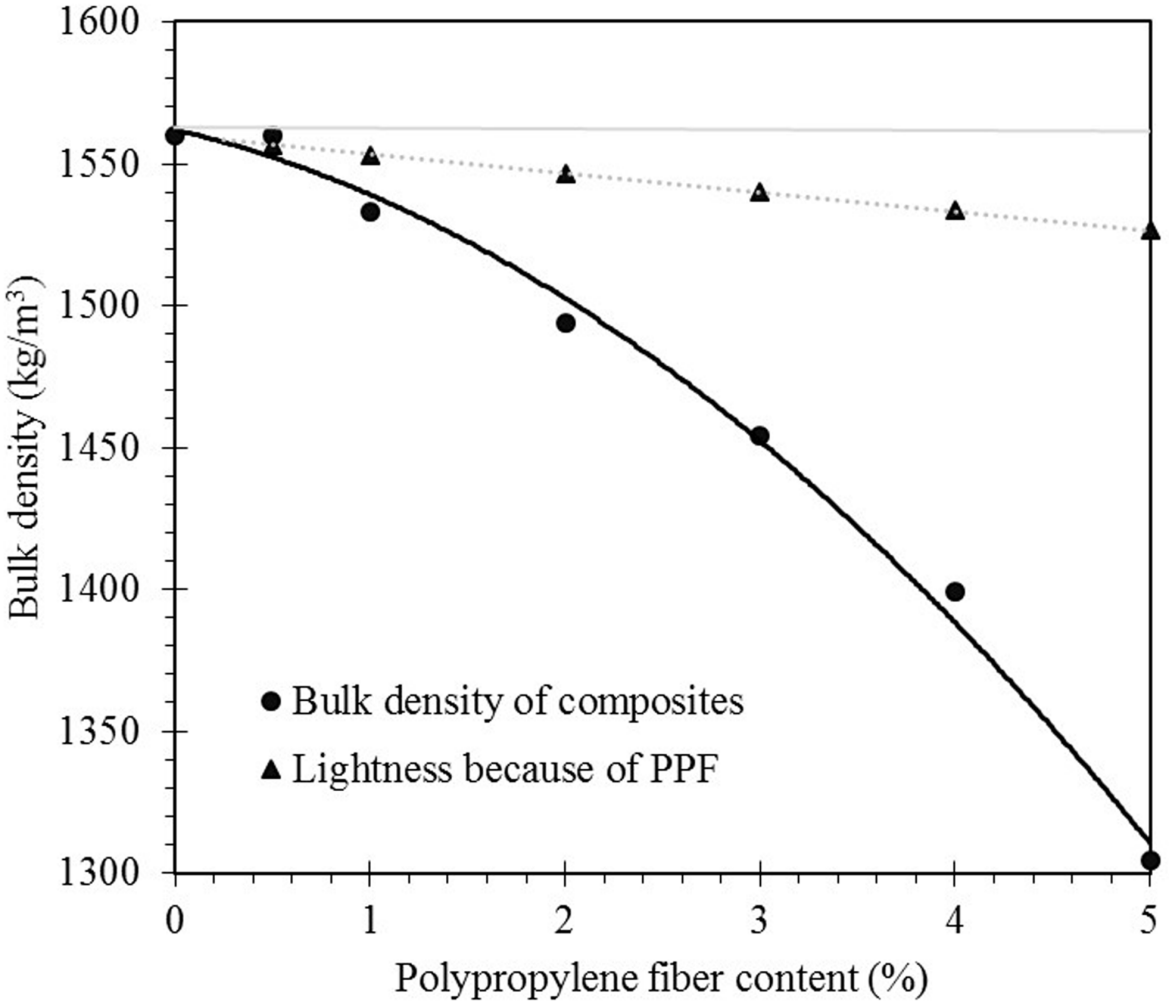
Effect of PPF content on density reduction of PPF fly ash based geopolymer.

Fig 8 indicated that the density of PPF fly ash based geopolymer decreased by increasing of PPF content of the mixture and the theoretical weight loss due to lower specific gravity of the polypropylene. About 20% reduction of density was observed in 5% PPF content matrix compared to the specimen without PPF content that attributed to the high porosity of the matrix due to difficulties of compaction concomitant with lower specific gravity of the fibers. However, from the rule of mixture method it can be concluded that addition of fibers is not a dominant factor in reducing the density. Therefore, the pores which were trapped among the clusters of fiber in fresh states govern the reduction of density in hardened matrix.

### 3.5. Shrinkage effects

**Fig 9:**
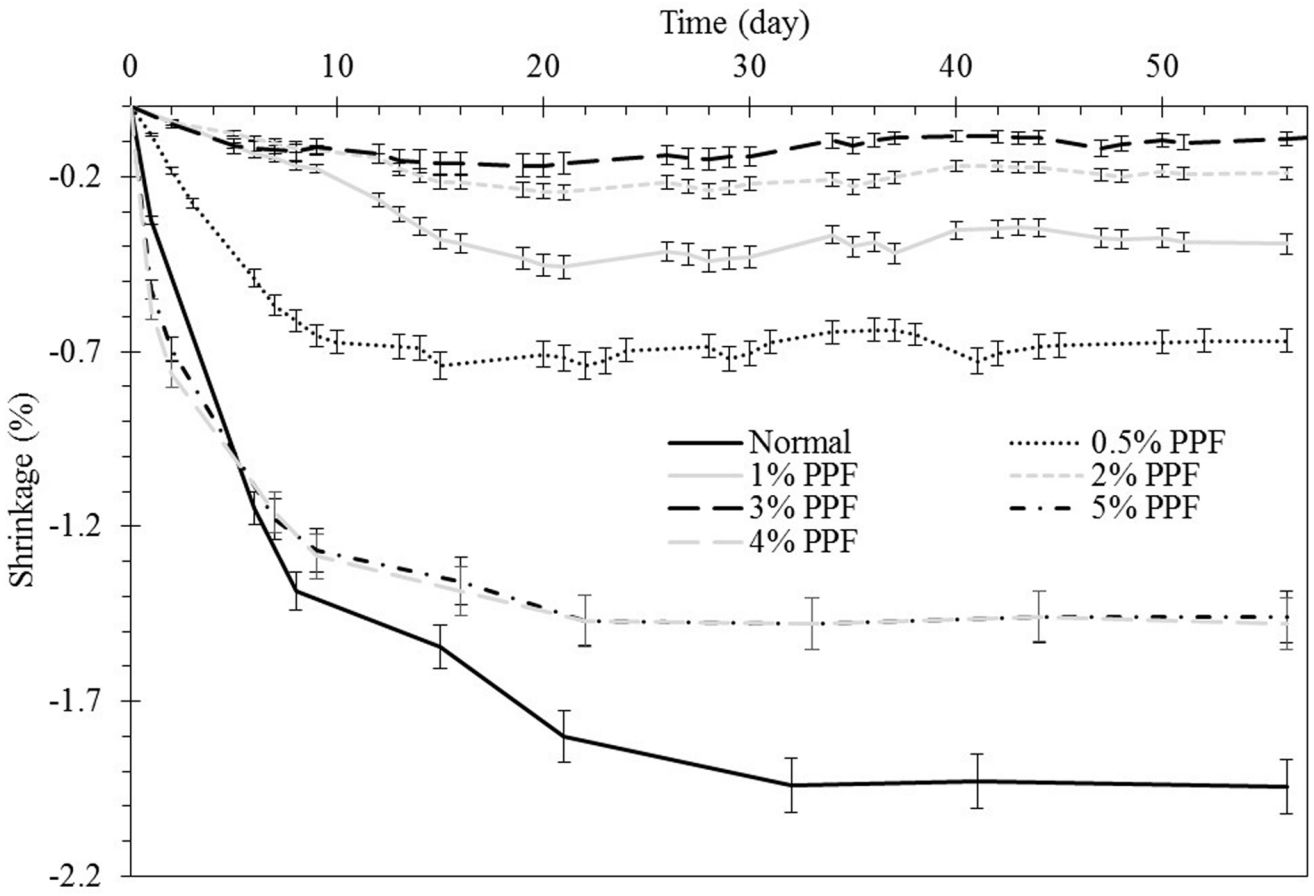
PPF content effect on controlling the shrinkage of fly ash based geopolymer.

Dry shrinkage and cracking of geopolymers was reported as a result of generation of high capillary pressures of wet and dry part of a micropore network which caused micro crack propagation [11, 34]. As observed in Fig 9, addition of PPF in a small volume fraction from 0.5% to 3% reduced the shrinkage of the geopolymer composites. This reduction might be attributed to two major effects. First, shrinkage was restrained because of promoted tensile stresses of the matrix attributed to clamping pressure and frictional bond in geopolymer matrix and fiber interface; therefore, a part of shrinkage energy was nullified in friction of the interfaces [35]. In addition, PPF provides bridging forces across cracks and retards their growth. However, increasing the fiber content to 4% to 5% had an adverse effect on shrinkage of geopolymer composites and increased it significantly. This originates from the high porosity of composite as discussed in section 3.4; the increase in pore volume result in retention of moisture which contributed to the acceleration of shrinkage [36].

**Fig 10:**
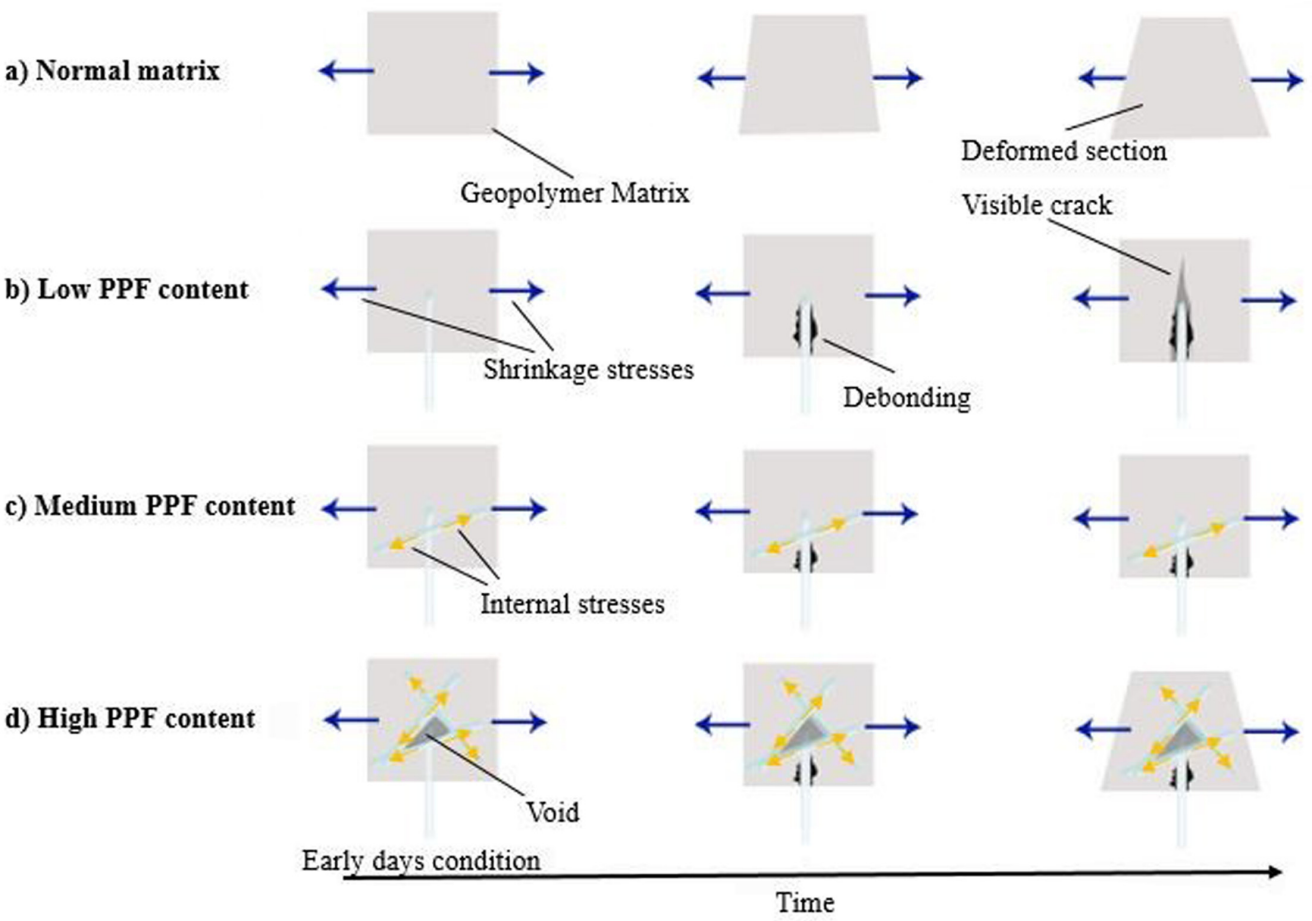
Schematic shrinkage effect on PPF fly ash based geopolymer composites.

In addition to trends of shrinkage variation which were measured by demec gauge, different types of visual specimen deformation were observed based on the fiber content. Fig 10 shows the schematic image of shrinkage effect on different composites. As indicated, the geopolymer composite is categorized into four types including normal geopolymer matrix, low, medium and high PPF content fly ash based geopolymer composites. The normal geopolymer matrix without PPF content shows high shrinkage strain however the deformation type appeared as geometrical without any visible cracks in samples. Low PPF content matrix of 0.5% underwent shrinkage strain with high rate for about 7 days and then remained unchanged. Although a small amount of fiber in geopolymer paste could reduce shrinkage and remove the evidence of geometrical deformation, visible cracks appeared in the specimens parallel to their cross section. These cracks were because of the weak bonding between PPF and geopolymer matrix which cannot overcome the shrinkage stress. The weak contact originated from hydrophobic and smooth surface of PPF concomitant with non-polar C-C bonds of the polypropylene which inhibits adhesion to matrix, as shown in section 3.1 [37, 38]. Indeed, fibers which locate parallel to the main axis of specimen enhance the shrinkage resistance of the matrix due to a large interface contact area in the effective shrinkage direction; therefore they can overcome the stress. However, the weak contact of perpendicular fibers concomitant with their small interface contact and might not carry the shrinkage stress of the section. Since, in low fiber content specimens, the number of fibers at each section of the beam are fewer, and as the fiber orientation are definitely random, the potential of overcoming the accumulation of shrinkage stress was reduced at weak sections and the possibility of cracking increased. The medium fiber content samples including 1% to 3% control the shrinkage significantly which is the best in 3% addition of the PPF. Increasing the amount of fiber increased the sections’ number of fibers resulting in decrease in probability of localized poor distribution and increasing the uniformity of the composites. Thus, PPF is able to provide reinforcing mechanisms, arrest the matrix cracks and stabilize them before leading to unstable dimensions. Further increasing of the PPF content to 4% and 5% led to abundance of pores in the matrix as discussed in section 3.4. The porous structure of the matrix concomitant with the low Young's modulus of the fiber resulted in high shrinkage strain as observed in Fig 8. Although the shrinkage is high, abundance of PPF at the section controls the shrinkage cracking. Therefore, the samples are deformed geometrically without visible cracks.

### 3.6. Flexural properties

**Fig 11:**
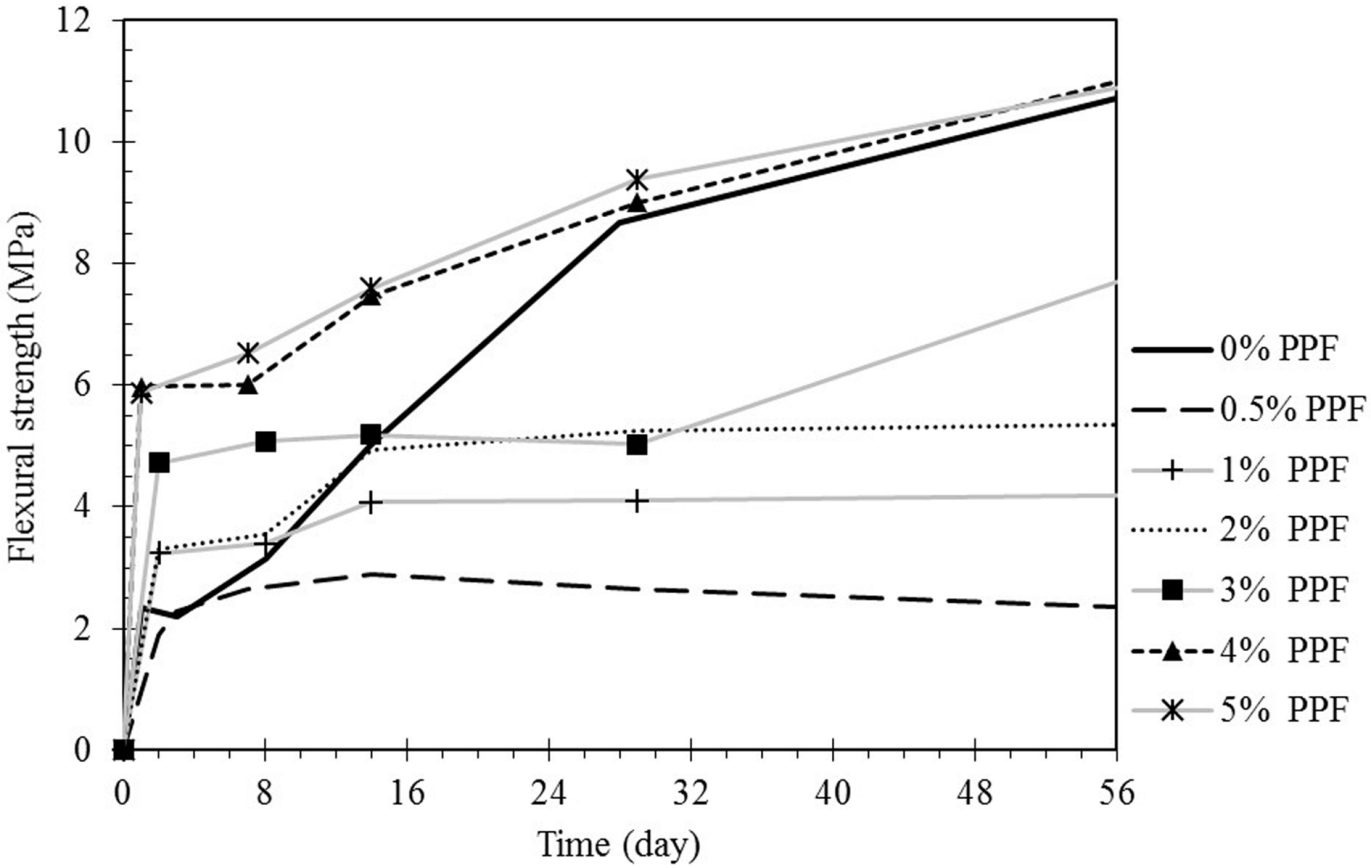
Flexural strength development of the PPF fly ash based geopolymer over time.

As indicated in Fig 11, addition of 1% PPF and above improves the early flexural strength of the matrix. However, it had an adverse effect in low fiber content specimens. At the early ages, the flexural strength of the matrix is still developing and much lower than the tensile strength of the fibers. Therefore, increasing the fiber content and bridging effect are the dominant factors in flexural properties enhancement. However, as discussed in section 3.5, in low fiber content matrixes, the potential of unrestricted defect propagation at interface of fibers and geopolymer paste is higher which result in reduction of ultimate capacity of the beam specimens.

The flexural strength of the geopolymer paste increased over time due to completion of the reaction. Although the geopolymer paste has more potential to carry the applied stress, over the age of 7 days shrinkage cracks appeared in low fiber content specimens (0.5%) parallel to the section of the beam; therefore, there was less resistance against the flexural load and stresses normal to cross sections. In medium fiber content specimens of 1%, 2% and 3%, the final strength of the matrix improved lightly compared to the early strength; in these cases the effects of paste hardening is nullified with defects on interface of the bonds and geopolymer due to shrinkage and weak fiber-matrix bond. It can be observed that the effect of paste hardening is dominant factor in development of the flexural strength in the high PPF content composites.

**Fig 12:**
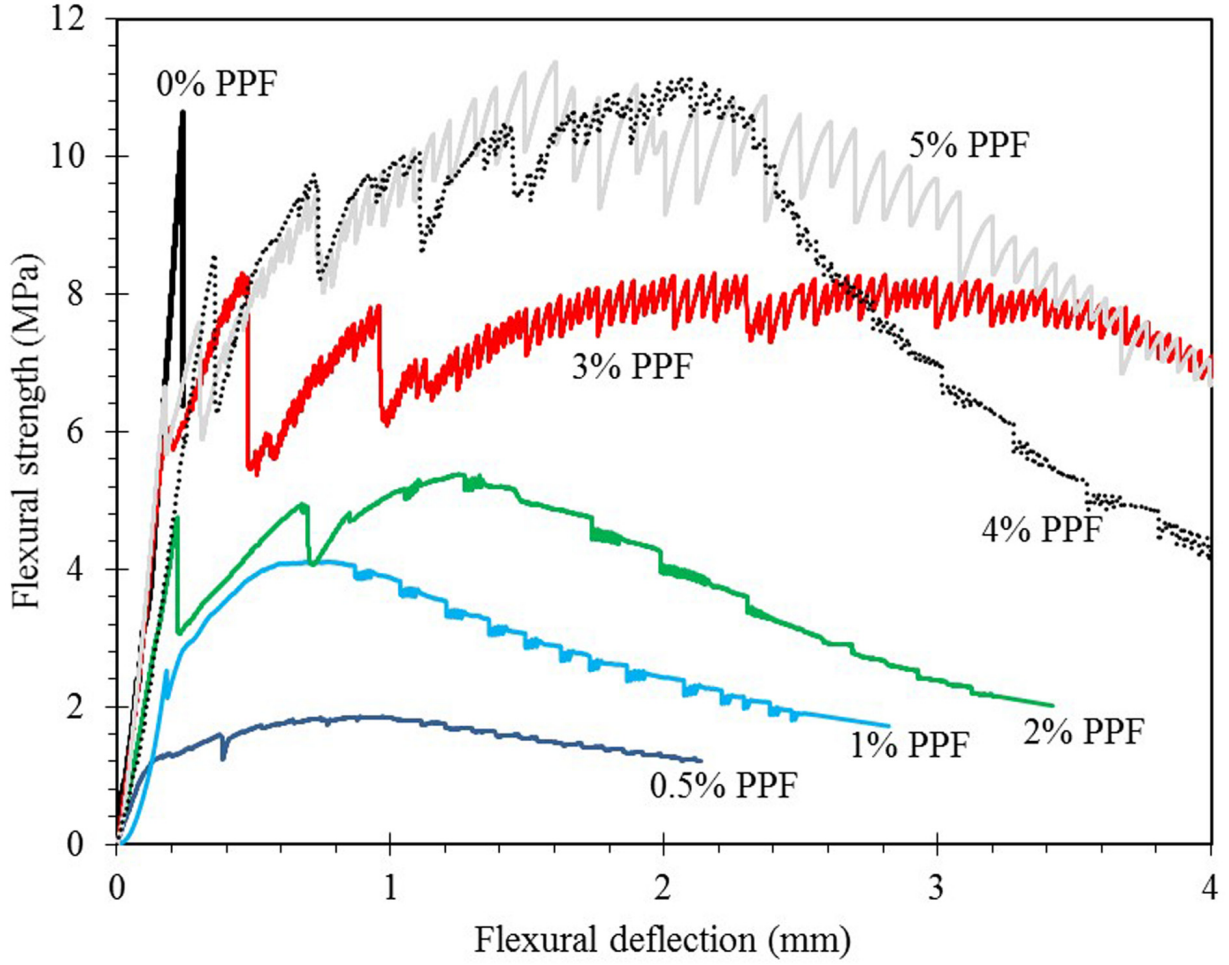
Flexural strength-deflection curve of PPF geopolymer matrix at 56 days.

**Fig 13:**
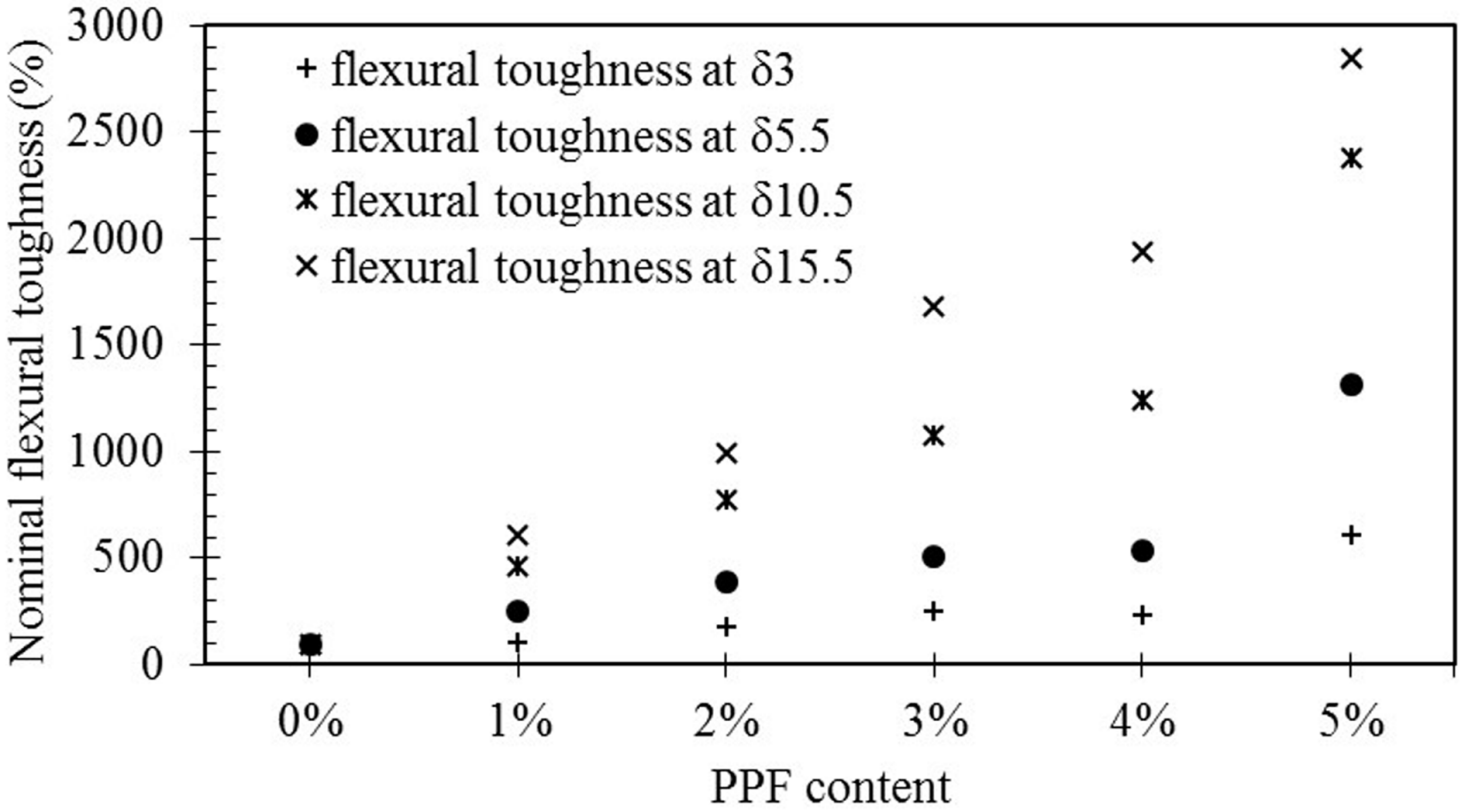
Nominal flexural toughness improvement of PPF fly ash based geopolymer composites at 56 days.

Fig 12 shows the flexural strength-deflection curves of the PPF geopolymer composites after 56 days. As observed, the hardened geopolymer matrix without PPF content behave very brittle and the strength of its first crack is quite high compared to the specimens with fibers. In contrast, addition of PPF fiber increase the shrinkage defects and additional porosity to the matrix leading to decrease in effective cross section; therefore, nullifying the flexural strength of the geopolymer matrix itself and reducing the stress at which the first micro crack of the composite commenced. Similar behavior was observed in other cementitious composites and increase in porosity cased a reduction of flexural strength [39]. However, fiber restricts the widening and propagating of crack. Therefore, it improves the energy absorption capability of the composite. Because of this, the composite depicted a strain-hardening beyond the first crack point and made the material more ductile. Fig 13 indicated the variation of nominal flexural toughness based on the δ, flexural displacement of first crack, and PPF content. The increase in nominal flexural toughness increased by increasing the PPF content. The nominal flexural toughness corresponding to δ_15.5_ improved noticeably. This enhancement is originated from the slow rate softening of the material leading to significant energy absorption.

**Fig 14:**
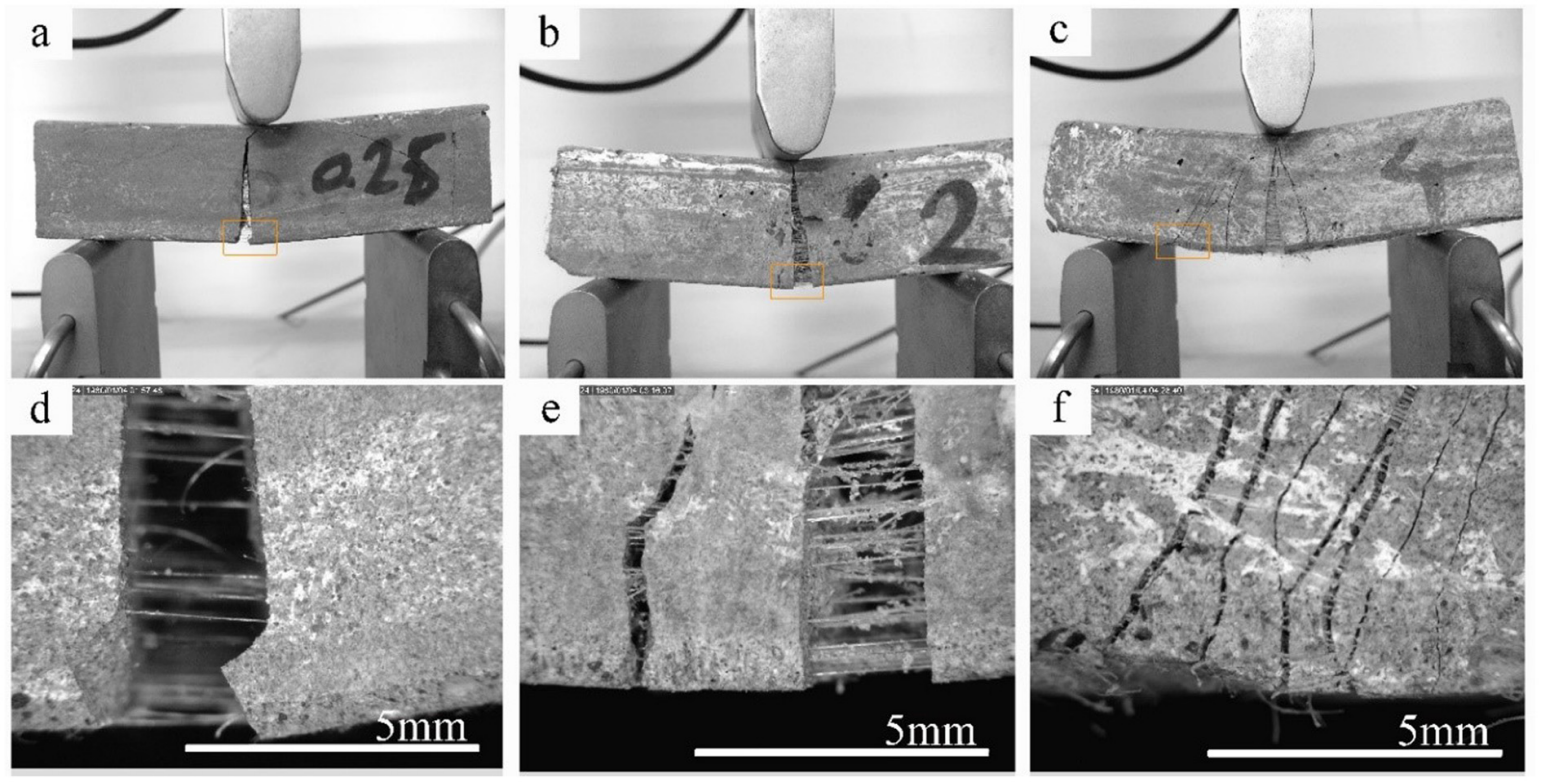
Fracture mode and toughening mechanism of PPF reinforced fly ash based geopolymer.

Fig 14a–14c showed the failure mode of the low, medium and high PPF content specimens, respectively. As indicated in Fig 12-e, the material with low fiber content experienced a sharp brittle crack while specimens with more fibers had more than one crack at their failure (Fig 14e and 14f); once a crack formed in the specimens, a strength drop was observed in flexural strength-deflection curve and bending stress was transferred to the fibers. The bridging effect of the fibers prevent the sudden failure of specimens at the crack region and transfer the stress to the other parts. Since the tensile strength of the polypropylene is high, generally they resist by their bridging and are released by pull out action. Once the stress in another section has surpassed capacity, a new crack is formed and the same procedure repeated. It is notable that one crack is the active though the loading period and failure occurs in that section. Fig 12-f indicated that although many secondary cracks formed at different sections of the specimen, one crack is the active which will widen with higher rate though the test.

### 3.7. Field emission scanning electron microscopy

**Fig 15:**
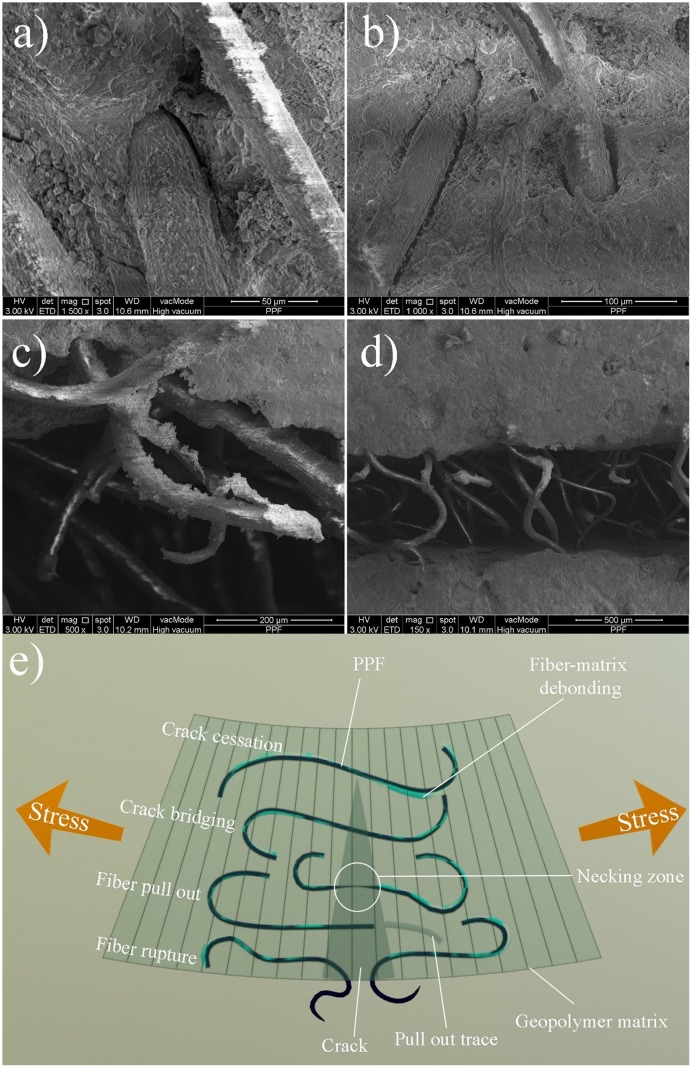
(a-d) FESEM images and (e) schematic mechanism of PPF and geopolymer matrix interaction.

Fig 15a and 15b shows the FESEM images of the PPF interaction with FA based geopolymer composite before subjecting to an external load. The PPF composite, indicates a weak interfacial transition zone (ITZ) in the PPF—geopolymer matrix interface at 56 days which is related to the large shrinkage of the geopolymer paste as discussed in the section 3.3. There is approximately 5 μm distance between PPF and surface of the geopolymer matrix. Furthermore, some micro-cracks were also formed in geopolymer at the ITZ zone. Fig 15c and 15d show the condition of the PPF at crack zone when the specimen was subjected to a three-point bending load. The PPF might deformed and lengthen, ruptured or pull out because of the applied stress at fiber section in a crack zone. If the fiber has enough clamping force and friction to overcome pull out, it can resist the load until it ruptured; otherwise, the smooth surface and weak interfacial fiber-matrix bond resulted in pull out the fiber without a considerable resistance against the applied load. As observed in the Fig 12, the first cracking load of the fiber reinforced composites is lower than that of the normal geopolymer specimens however after the first crack still they are able to carry loads. This extra resistance is because of the energy absorption of the fibers to deform [40]. Fig 15d shows the deformed condition of the fibers at a crack zone after unloading of the specimens. The mechanism of the PPF on geopolymer matrix is schemed in Fig 15e.

### 3.8 Compressive strength

**Fig 16:**
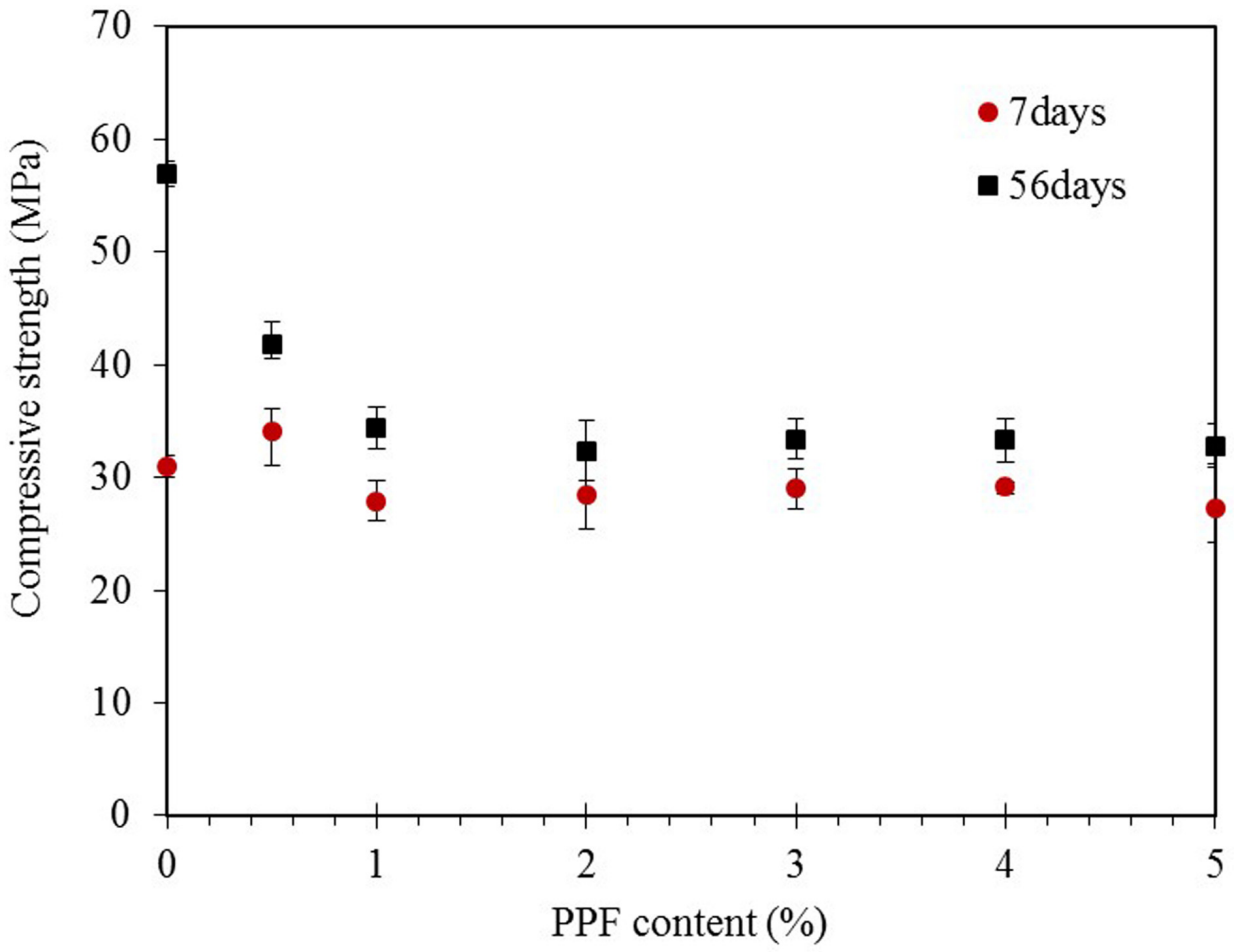
Compressive strength variation of the PPF reinforced fly ash based geopolymer composites in 7 and 56 days.

The compressive strength variation of the PPF reinforced fly ash based geopolymer composites was presented in Fig 16 from days 7 and 56. As shown, there is negligible difference between compressive strength of the specimens at the early age of curing (7 days). At this period, compressive strength of the matrixes are still developing while porous defects in PPF content samples can be overcome by the shear reinforcing of the fibers; thus, compressive strength of the matrix remained relatively unaffected. However, the geopolymer matrix gained strength over time and improved about 84%. The strength of the fiber reinforced specimens did not follow the same trend as hardened geopolymer matrix without fiber content; excluding low fiber content specimens of 0.5%, others enhanced about 17%; this cessation of compressive strength development might be attributed to shrinkage defects due to the weak contact interface of fibers and geopolymer matrix as discussed in previous sections, resulting in easy microcrack formation through the specimens and decreasing the shear stress resistance [41]. Previous works also reported the same trend of compressive strength gain in that PPF reduced the rate of compressive strength development [16, 27]. Thus, although the geopolymer matrix itself is strong locally, it does not behave strong once it is in interaction with PPF as a composite.

## Conclusion

This study evaluated fly ash based geopolymer reinforced by polypropylene fibers (PPF) from the aspects of shrinkage variation, fresh and mechanical properties. Based on our experiments, the following conclusions were drawn:

The workability of the composites is reduced significantly by increasing the percentages of fiber inside because of higher shear resistance to flow. Moreover, setting time was affected and compressibility of the materials increased.

Shrinkage of the composite can be controlled based on the fiber content which was the best for 3% addition of PPF into the geopolymer matrix; moreover, shrinkage variation plays important role in the mechanical properties of the PPF reinforced fly ash based geopolymer composites. Based on the fiber content, shrinkage effects might appear in the form of geometrical deformation with or without visible cracks.

The mechanical properties of the composites are governed by the strength development of the geopolymer matrix itself. It was observed that both compressive and flexural strength of the pure geopolymer specimens, without PPF content, was increased by time. However, incorporation of the PPF into the geopolymer nullified the effects of geopolymer matrix strengthening because of weak fiber-matrix interfacial contact and breaking the geopolymer bonds.

Although, the presence of polypropylene fibers in fly ash based geopolymer matrix did not lead to increase in the flexural strength, the post-peak load carrying capacity was enhanced due to toughened enhanced mechanism of fibers; hence the flexural toughness or energy absorption of the material was improved.

## References

[1] MACKENZIE K, WELTER M. Geopolymer (aluminosilicate) composites: synthesis, properties and applications. Advances in Ceramic Matrix Composites. 2014:445.

[2] Bakharev T. Geopolymeric materials prepared using Class F fly ash and elevated temperature curing. Cement and Concrete Research. 2005;35(6):1224–32.

[3] Davidovits J. Geopolymer Chemistry and Applications. France: Institut Geopolymere, Saint-Quentin; 2008.

[4] Tchadjié L, Djobo J, Ranjbar N, Tchakouté H, Kenne B, Elimbi A, et al Potential of using granite waste as raw material for geopolymer synthesis. Ceramics International. 2016;42(2):3046–55.

[5] Sakulich AR. Reinforced geopolymer composites for enhanced material greenness and durability. Sustainable Cities and Society. 2011;1(4):195–210.

[6] Duxson P, Fernández-Jiménez A, Provis JL, Lukey GC, Palomo A, van Deventer JSJ. Geopolymer technology: the current state of the art. J Mater Sci. 2007;42(9):2917–33.

[7] Ranjbar N, Talebian S, Mehrali M, Kuenzel C, Metselaar HSC, Jumaat MZ. Mechanisms of interfacial bond in steel and polypropylene fiber reinforced geopolymer composites. Composites Science and Technology. 2016;122:73–81.

[8] Alomayri T, Shaikh FUA, Low IM. Synthesis and mechanical properties of cotton fabric reinforced geopolymer composites. Composites Part B: Engineering. 2014;60(0):36–42.

[9] Dias DP, Thaumaturgo C. Fracture toughness of geopolymeric concretes reinforced with basalt fibers. Cement and Concrete Composites. 2005;27(1):49–54.

[10] Pan Z, Sanjayan JG, Rangan BV. Fracture properties of geopolymer paste and concrete. Magazine of concrete research. 2011;63(10):763–71.

[11] Kuenzel C, Vandeperre LJ, Donatello S, Boccaccini AR, Cheeseman C. Ambient Temperature Drying Shrinkage and Cracking in Metakaolin‐Based Geopolymers. Journal of the American Ceramic Society. 2012;95(10):3270–7.

[12] Zuhua Z, Xiao Y, Huajun Z, Yue C. Role of water in the synthesis of calcined kaolin-based geopolymer. Applied Clay Science. 2009;43(2):218–23.

[13] Ridtirud C, Chindaprasirt P, Pimraksa K. Factors affecting the shrinkage of fly ash geopolymers. Int J Miner Metall Mater. 2011;18(1):100–4.

[14] Perera D, Uchida O, Vance E, Finnie K. Influence of curing schedule on the integrity of geopolymers. J Mater Sci. 2007;42(9):3099–106.

[15] Ranjbar N, Mehrali M, Alengaram UJ, Metselaar HSC, Jumaat MZ. Compressive strength and microstructural analysis of fly ash/palm oil fuel ash based geopolymer mortar under elevated temperatures. Construction and Building Materials. 2014;65:114–21.

[16] Puertas F, Amat T, Fernández-Jiménez A, Vázquez T. Mechanical and durable behaviour of alkaline cement mortars reinforced with polypropylene fibres. Cement and Concrete Research. 2003;33(12):2031–6.

[17] Ranjbar N, Mehrali M, Mehrali M, Alengaram UJ, Jumaat MZ. Graphene nanoplatelet-fly ash based geopolymer composites. Cement and Concrete Research. 2015;76(0):222–31.

[18] Davidovits J. Geopolymers: Inorganic polymeric new materials. Journal of Thermal Analysis. 1991;37:1633–56.

[19] Alzeer M, MacKenzie KJ. Synthesis and mechanical properties of new fibre-reinforced composites of inorganic polymers with natural wool fibres. J Mater Sci. 2012;47(19):6958–65.

[20] He P, Jia D, Lin T, Wang M, Zhou Y. Effects of high-temperature heat treatment on the mechanical properties of unidirectional carbon fiber reinforced geopolymer composites. Ceramics International. 2010;36(4):1447–53.

[21] Bernal S, De Gutierrez R, Delvasto S, Rodriguez E. Performance of an alkali-activated slag concrete reinforced with steel fibers. Construction and Building Materials. 2010;24(2):208–14.

[22] Yunsheng Z, Wei S, Zongjin L, Xiangming Z, Eddie, Chungkong C. Impact properties of geopolymer based extrudates incorporated with fly ash and PVA short fiber. Construction and Building Materials. 2008;22(3):370–83.

[23] Alhozaimy A, Soroushian P, Mirza F. Mechanical properties of polypropylene fiber reinforced concrete and the effects of pozzolanic materials. Cement and Concrete Composites. 1996;18(2):85–92.

[24] Aulia TB. Effects of polypropylene fibers on the properties of high-strength concretes. Institutes for Massivbau and Baustoffechnologi, University Leipzig, Lacer. 2002;(7).

[25] BRE. Constructing the future. Design Build.2000.

[26] Richardson AE. Compressive strength of concrete with polypropylene fibre additions. Structural survey. 2006;24(2):138–53.

[27] Zhang Z-h, Yao X, Zhu H-j, Hua S-d, Chen Y. Preparation and mechanical properties of polypropylene fiber reinforced calcined kaolin-fly ash based geopolymer. Journal of Central South University of Technology. 2009;16:49–52.

[28] Ranjbar N, Mehrali M, Behnia A, Alengaram UJ, Jumaat MZ. Compressive strength and microstructural analysis of fly ash/palm oil fuel ash based geopolymer mortar. Materials & Design. 2014;59:532–9.

[29] ASTM. C1437-13 Standard Test Method for Flow of Hydraulic Cement Mortar. 2013.

[30] ASTM. C191-13 Standard Test Methods for Time of Setting Hydraulic Cement by Vicat Needle. 2013.

[31] ASTM. C20 Standard Test Methods for Apparent Porosity, Water Absorption, Apparent Specific Gravity, and Bulk Density of Burned Refractory Brick and Shapes by Boiling Water. 2015.

[32] ASTM. C1018-97 Standard Test Method for Flexural Toughness and First-Crack Strength of Fiber-Reinforced Concrete (Using Beam With Third-Point Loading) 2006.

[33] Morent R, De Geyter N, Leys C, Gengembre L, Payen E. Comparison between XPS‐and FTIR‐analysis of plasma‐treated polypropylene film surfaces. Surface and Interface Analysis. 2008;40(3‐4):597–600.

[34] Scherer GW. Theory of Drying. Journal of the American Ceramic Society. 1990;73(1):3–14.

[35] Stang H. Significance of shrinkage-induced clamping pressure in fiber-matrix bonding in cementitious composite materials. Advanced Cement Based Materials. 1996;4(3–4):106–15.

[36] Silva FdA, Filho RDT, Filho JdAM, Fairbairn EdMR. Physical and mechanical properties of durable sisal fiber—cement composites. Construction and Building Materials. 2010;24(5):777–85.

[37] López-Buendía AM, Romero-Sánchez MD, Climent V, Guillem C. Surface treated polypropylene (PP) fibres for reinforced concrete. Cement and Concrete Research. 2013;54(0):29–35.

[38] Carstens P, Marais S, Thompson C. Improved and novel surface fluorinated products. Journal of Fluorine Chemistry. 2000;104(1):97–107.

[39] Ranjbar N, Behnia A, Alsubari B, Birgani PM, Jumaat MZ. Durability and mechanical properties of self-compacting concrete incorporating palm oil fuel ash. Journal of Cleaner Production. 2016;112:723–30.

[40] Singh S, Shukla A, Brown R. Pullout behavior of polypropylene fibers from cementitious matrix. Cement and Concrete Research. 2004;34(10):1919–25.

[41] Bazant ZP, Ozbolt J. Compression failure of quasibrittle material: Nonlocal microplane model. Journal of engineering mechanics. 1992;118(3):540–56.

